# Identification of gluten T cell epitopes driving celiac disease

**DOI:** 10.1126/sciadv.ade5800

**Published:** 2023-01-25

**Authors:** Markéta Chlubnová, Asbjørn O. Christophersen, Geir Kjetil F. Sandve, Knut E.A Lundin, Jørgen Jahnsen, Shiva Dahal-Koirala, Ludvig M. Sollid

**Affiliations:** ^1^ KG Jebsen Coeliac Disease Research Center, University of Oslo, Oslo, Norway.; ^2^ Institute of Clinical Medicine, University of Oslo, Oslo, Norway.; ^3^ Department of Rheumatology, Dermatology, and Infectious Diseases, Oslo University Hospital, Oslo, Norway.; ^4^ Department of Informatics, University of Oslo, Oslo, Norway.; ^5^ Department of Gastroenterology, Oslo University Hospital Rikshospitalet, Oslo, Norway.; ^6^ Department of Gastroenterology, Akershus University Hospital, Lørenskog, Norway.; ^7^ Department of Immunology, Oslo University Hospital, Oslo, Norway.

## Abstract

CD4^+^ T cells specific for cereal gluten proteins are key players in celiac disease (CeD) pathogenesis. While several CeD-relevant gluten T cell epitopes have been identified, epitopes recognized by a substantial proportion of gluten-reactive T cells remain unknown. The identification of such CeD-driving gluten epitopes is important for the food industry and in clinical settings. Here, we have combined the knowledge of a distinct phenotype of gluten-reactive T cells and key features of known gluten epitopes for the discovery of unknown epitopes. We tested 42 wheat gluten–reactive T cell clones, isolated on the basis of their distinct phenotype and with no reactivity to known epitopes, against a panel of synthetic peptides bioinformatically identified from a wheat gluten protein database. We were able to assign reactivity to 10 T cell clones and identified a 9-nucleotide oligomer core region of five previously uncharacterized gliadin/glutenin epitopes. This work represents an advance in the effort to identify CeD-driving gluten epitopes.

## Teaser

Knowledge-based strategy identified five previously uncharacterized wheat gluten T-cell epitopes implicated in celiac disease.

## INTRODUCTION

Celiac disease (CeD) is an autoimmune-like disorder mediated by CD4^+^ T cell response to deamidated gluten peptides that are selectively presented by disease-predisposing human leukocyte antigen (HLA) molecules (HLA-DQ2.5, HLA-DQ2.2, and HLA-DQ8) (*1*). Gluten is a collective term for glutamine- and proline-rich proteins of wheat, barley, and rye. Gluten proteins of wheat are termed gliadins (α/β/ω subtypes) and glutenins (low molecular weight/high molecular weight subtypes), whereas in barley, they are termed hordeins and, in rye, secalins (*2*). The only available treatment for CeD is the elimination of gluten from the diet.

The gluten proteome is extremely complex, consisting of hundreds of similar proteins (*3*, *4*). However, only a small fraction of all possible gluten fragments is recognized by gluten-reactive CD4^+^ T cells of patients with CeD, suggesting a strict selection of T cell epitopes. Three main selection factors have been identified, including (i) proteolytic stability to gastrointestinal digestion, (ii) substrate affinity to the enzyme transglutaminase 2 (TG2) mediating deamidation, and (iii) selectivity in binding to disease-predisposing HLA molecules (*5*, *6*). In particular, high proline (P) content is crucial for rendering peptides resistant to proteolytic degradation (*5*, *7*). Furthermore, TG2 preferentially deamidates glutamine (Q) residues in the sequence context “QXP” and “QXX(F/Y/W/M/L/I/V)” (*8*, *9*). Last, the CeD-associated HLA-DQ types prefer negatively charged anchor residues at specific positions within the 9-nucleotide oligomer–binding core region (i.e., HLA-DQ2.5/DQ2.2 at positions P4, P6, and P7, and HLA-DQ8 at positions P1 and P9) (*10*). Hitherto, 27 HLA-DQ2.5–restricted gluten epitopes have been identified, of which 4 epitopes derived from wheat (DQ2.5-glia-α1a, DQ2.5-glia-α2, DQ2.5-glia-ω1, and DQ2.5-glia-ω2) and 1 from barley (DQ2.5-hor-3) are considered immunodominant (*11*, *12*).

Despite more than two decades of extensive work, many gluten T cell epitopes remain unidentified. Among gluten-reactive T cell clones (TCCs) from patients with CeD, 30 to 50% were found to be unreactive to known epitopes (*13*, *14*). Characterizing these unknown epitopes will be important for proper monitoring of gluten-free food (*15*, *16*), for diagnosis and monitoring of CeD by detection of gluten-specific T cells with HLA-DQ:gluten tetramers (*17*, *18*), and for generation of grains that are nonharmful to patients with CeD (*19*–*21*). The starting point for T cell epitope characterization is access to CeD-relevant TCCs with unknown epitope specificity. In a recent study (*22*), we described a method to isolate T cells solely on the basis of distinct phenotypic markers (CD3^+^, CD4^+^, CD127^−^, PD-1^+^, CD161^+^, ICOS^+^, CD39^+^, and CXCR3^+^) that are hallmarks of the gluten-specific T cells (*23*). By generating TCCs from such phenotypically distinct T cells, we found that more than 65% of these TCCs were indeed gliadin/glutenin reactive (*22*). Here, we identified yet uncharacterized T cell epitopes by testing phenotypically distinct gluten-reactive TCCs with unknown epitope specificity against peptides representing potential HLA-DQ2.5–restricted epitopes identified by knowledge-based strategies.

## RESULTS

### Phenotypically isolated gluten-reactive T cells as tools for epitope discovery

**Fig. 1.**
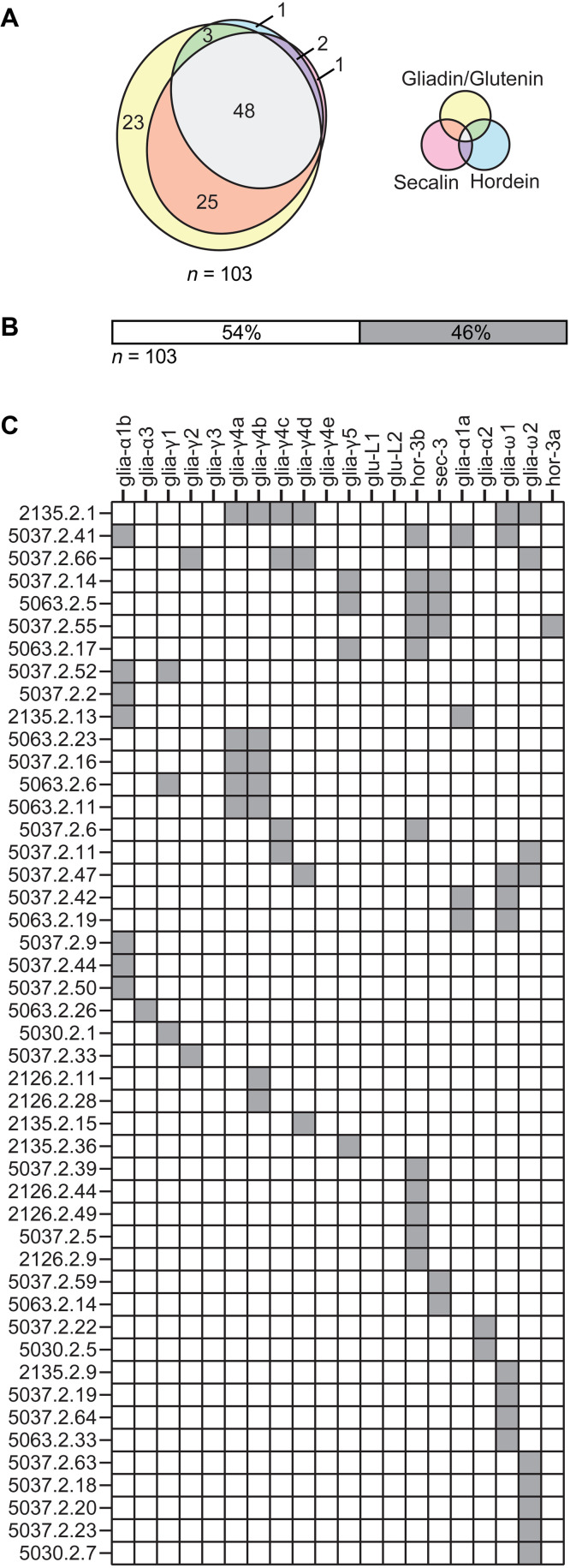
Grain reactivity and epitope specificity of gluten-reactive TCCs. (**A**) From 179 TCCs that carried the phenotype typical of gluten-specific T cells in CeD and that stained negative with HLA-DQ:gluten tetramers, we identified 103 TCCs that were reactive to chymotrypsin-digested and TG2-treated gliadin/glutenin, hordein, or secalin. The Venn diagram depicts the number of TCCs reactive to the respective gluten proteins. All gluten proteins were tested at a concentration of 10 μg. The TCCs that were not reactive to any of the digests are not shown. (**B**) Frequency of the gluten-reactive TCCs recognizing known (filled box) or unknown epitopes (*n* = 103) in T cell proliferation assays. (**C**) Epitope specificity of the gluten-reactive TCCs. The individual TCCs are indicated by numbers and the peptides are indicated by epitope names. Only TCCs that were reactive to at least one tested peptide are displayed (47 of 103) (filled boxes). All peptides were tested in T cell proliferation assays at 10 μM concentration. The TCCs were considered reactive if the stimulation index (SI) was higher than 1.8. This screening experiment that aims to select for informative TCCs was performed once in triplicate.

In this study, we selected 179 TCCs generated in a previous study (*22*) that carried the phenotype typical to gluten-specific T cells in CeD and did not stain with a mixture of HLA-DQ:gluten tetramers representing five immunodominant gluten epitopes. We tested these TCCs against the deamidated digests of both gliadin/glutenin, hordein, and secalin and found that 103 TCCs displayed reactivity. Almost half of these TCCs were cross-reactive to all three deamidated digests, which suggests that they recognized epitopes shared between wheat, barley, and rye (Fig. 1A and table S1). Only few TCCs were uniquely reactive to secalin and/or hordein (Fig. 1A and table S1). Next, we tested the epitope specificity of these 103 TCCs against a panel of synthetic peptides representing known HLA-DQ2.5 T cell epitopes (table S2). We found that 46% (47 of 103) of these TCCs were reactive to at least one known HLA-DQ2.5 T cell epitope (Fig. 1B and table S1). Some TCCs classified as negative for HLA-DQ:gluten tetramer staining made a response in the T cell proliferation assay to peptides represented in the mixture of HLA-DQ:gluten tetramers. On restaining with HLA-DQ:gluten tetramers, such TCCs had a slightly higher mean fluorescence intensity (MFI) than negative control TCCs but a much lower MFI than TCCs originally sorted as tetramer positive. Titrating the peptide antigens in a T cell proliferation assay, we observed that such TCCs made positive responses only to high concentration of antigen (fig. S1), suggesting that lower affinities of T cell receptors could explain the behavior of these TCCs. Together, we identified 56 TCCs that did not make a response to any known gluten peptide epitope. All these 56 TCCs were found to be reactive to gliadin/glutenin alone or in combination with secalin and/or hordein, indicating that the epitopes recognized by these TCCs are present in the proteome of wheat. These TCCs were thus used as tools to identify yet uncharacterized epitopes in gliadin/glutenin (*Triticum aestivum*) (table S1).

### Strategies to identify potential candidates for gliadin/glutenin epitopes

We designed two strategies based on characteristic shared features of known gliadin/glutenin epitopes to identify yet uncharacterized epitopes. Using these strategies, we searched the GluPro I database, a manually curated proteome database containing unique gliadin/glutenin peptide sequences of *T. aestivum* (*3*), for the presence of candidate 9-nucleotide oligomer sequences.

#### Strategy 1—Simple algorithm database search

We identified a motif, P-[FQ]-P-Q-[PLQ]-[PQ]-[FLQY]-P-[FQWY], predominantly present in the native peptide sequences of four immunodominant gliadin/glutenin epitopes—DQ2.5-glia-α1a (PFPQPQLPY), DQ2.5-glia-α2 (PQPQLPYPQ), DQ2.5-glia-ω1 (PFPQPQQPF), and DQ2.5-glia-ω2 (PQPQQPFPW)—and searched for this motif in the GluPro I database by using the software tool ScanProsite (https://prosite.expasy.org). This search generated a finite list of 17 unique peptide sequences in no particular order, of which 5 were known gliadin/glutenin epitopes.

#### Strategy 2—Advanced algorithm database search

Next, we developed a more advanced algorithm based on characteristic features of all known HLA-DQ2.5–restricted gliadin/glutenin epitopes (*n* = 17) (table S2), namely, TG2 deamidation preferences, amino acid composition, and immunodominance. Specifically, we designed a mixture model composed of three hierarchical hidden Markov models, where each included a position weight matrix–like subpath, to predict and score candidate 9-nucleotide oligomer sequences. These position weight matrices were created on the basis of the following parameters: (i) the presence of deamidation patterns “QXP” and “QXX” (where X can be any amino acid) at positions 4, 6, or both of the 9-nucleotide oligomer amino acid sequence, where a separate position weight matrix of amino acid preference was estimated for each pattern based on the amino acid composition of deamidation patterns of known gliadin/glutenin epitopes, and (ii) amino acid preference in the 9-nucleotide oligomer core sequences of the known gliadin/glutenin epitopes outside the deamidation patterns, as well as an arbitrarily weighted preference of such amino acids present in the four immunodominant gliadin epitopes (table S2). In addition, the algorithm did not allow generation of 9-nucleotide oligomer sequences with more than four consecutive glutamine residues (QQQQ) and the presence of two consecutive prolines in the deamidation pattern (QPP).

Two variants of strategy 2 were used. In strategy 2A, the predicted 9-nucleotide oligomer sequences were scanned against the GluProI database to generate a list of top scoring 100 peptide sequences of potential gliadin/glutenin epitopes (table S3). In strategy 2B, scores corresponding to each 9-nucleotide oligomer were multiplied with the number of hits within the GluPro I database to generate the second list of top 100 peptide sequence of potential gliadin/glutenin epitopes (table S3). Last, we selected the top 10 scoring 9-nucleotide oligomer peptide sequences of potential gliadin/glutenin epitopes from each variant of strategy 2 (2A and 2B), where five sequences were overlapping (Table 1).

**Table 1.** Peptide sequences of potential gliadin/glutenin epitopes generated by strategies 1 and 2 (A and B). Column 1 shows the list of 9-nucleotide oligomer peptide sequences of the potential gliadin/glutenin epitopes identified by strategies 1 and 2 (A and B). The potential TG2 deamidation sites are underlined in the sequences. The cross marks in columns 2, 3, and 4 indicate the potential gliadin/glutenin epitopes identified by each strategy. Column 5 displays the sequence of peptides synthesized for testing where 9-nucleotide oligomer peptide sequences (boldfaced) are flanked with two native amino acids on both the N and C termini. The peptides were deamidated by TG2, and the potential TG2 deamidation sites are underlined in the sequence. Column 6 shows assigned corresponding peptide IDs of peptides tested in T cell proliferation assays.

Peptide-binding register (9-nucleotide oligomer)	Strategy			Sequences of 13-nucleotide oligomer peptides tested in T cell proliferation assay	Peptide ID of 13-nucleotide oligomer peptides tested in T cell proliferation assay	Peptide ID of 20-nucleotide oligomers reported in (*12*) that contain the 9-nucleotide oligomer sequence
	1	2A	2B			
PQPQQQLPQ	X	X		PF**PQPQQQLPQ**PQ	P2369	-
PQPQQPYPQ	X	X	X	PF**PQPQQPYPQ**PQ	P2368	R13, R28
PQPQQPFPQ	X	X	X	PY**PQPQQPFPQ**PQ	P2367	B07, R02, R17
PQPQPQYPQ	X			PY**PQPQPQYPQ**PQ	P2366	-
PQPQPQQPQ	X	X		SF**PQPQPQQPQ**QP	P2365	-
PQPQLQFPQ	X			PF**PQPQLQFPQ**QP	P2364	-
PQPQLPFPQ	X	X	X	PF**PQPQLPFPQ**QS	P2363	W06
PFPQQPYPQ	X	X	X	QQ**PFPQQPYPQ**QP	P2362	-
PFPQPQQPY	X	X		LQ**PFPQPQQPY**PQ	P2361	-
PFPQPQQPQ	X	X	X	QQ**PFPQPQQPQ**QP	P2360	W17, R23
PFPQPQLPF	X	X		QQ**PFPQPQLPF**PQ	P2359	W06
PFPQLQQPQ	X			QQ**PFPQLQQPQ**QP	P2358	W31
PQPQQPFPL		X		PF**PQPQQPFPL**RP	P2370	B02
QQPQLPFPQ			X	QP**QQPQLPFPQ**QP	P2371	W17, R24
QFPQPQQPQ			X	QQ**QFPQPQQPQ**QS	P2372	W35
PQPQQPQQQ			X	QF**PQPQQPQQQ**FP	P2373	-
PQPQLPYSQ			X	PF**PQPQLPYSQ**PQ	P2374	W08
IQPQQPFPQ			X	QF**IQPQQPFPQ**QP	P2375	W14, W25, W27, R03, R04, R05, R26, R29

Together, from strategies 1 and 2, we selected 18 unique peptide sequences of potential gliadin/glutenin epitopes, of which 9 were identified by both strategies (Table 1). These 18 peptide sequences were synthesized with two flanking amino acid residues at each end derived from the native sequences in GluProI database and then tested in T cell proliferation assays. Notably, the 9-nucleotide oligomer peptide–binding register of 11 candidate epitopes predicted by our strategies lie within previously reported 20-nucleotide oligomer peptides identified using peripheral blood T cells of gluten-challenged patients with CeD (Table 1) (*12*).

### Identification of f**ive previously uncharacterized** gliadin/glutenin epitopes

**Fig. 2.**
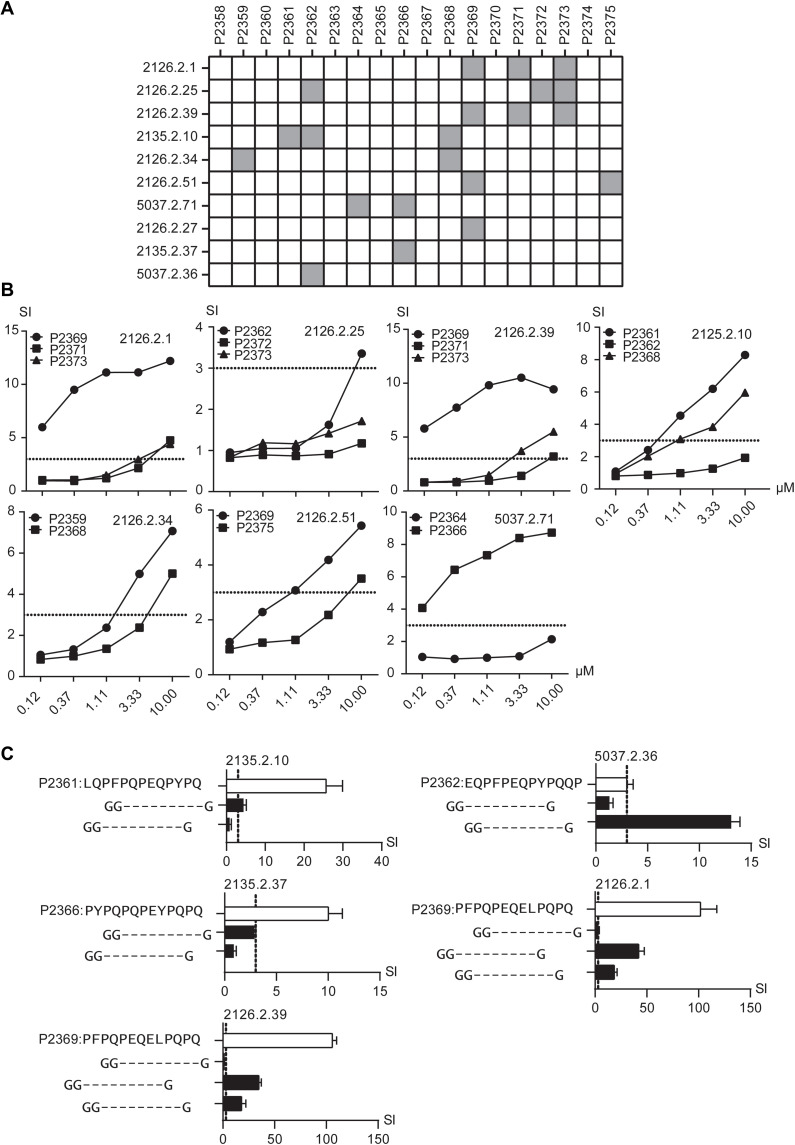
Identification of five previously uncharacterized gliadin/gluten epitopes. (**A**) Gliadin/glutenin-reactive TCCs (*n* = 42) were tested against a panel of 18 peptides in T cell proliferation assays. TCCs are indicated by numbers on the left and peptides are indicated by peptide IDs on the top (Table 1). TCCs (*n* = 10) that were reactive to at least one peptide are indicated as filled boxes. (**B**) Seven cross-reactive TCCs were examined in T cell proliferation assays with threefold titrated concentrations of the respective stimulatory peptides. (**C**) TCCs with previously defined stimulatory 13-nucleotide oligomer peptide sequence were tested against shorter peptides representing respective possible peptide-binding register variants flanked by two glycine residues at the N terminus and one glycine residue at the C terminus in a T cell proliferation assay. Only reactive TCCs are displayed. White bars represent original stimulatory 13-nucleotide oligomer sequences for each TCC, whereas black bars represent the reactivity to shorter peptides containing only a single possible candidate epitope sequence. TCCs reactive to at least one shorter peptide are displayed. Error bars in (C) represent SD. All peptides were tested at 10 μM concentration (unless stated otherwise) in triplicate. The TCCs were considered reactive if SI > 3 (stippled line). Results from one representative experiment of two are shown.

We tested the reactivity of 42 of 56 gliadin/glutenin-reactive TCCs with unknown epitope specificity (table S1) against a panel of 18 selected TG2-deamidated synthetic 13-nucleotide oligomer peptides representing the potential novel gliadin/glutenin epitopes (Table 1) in T cell proliferation assays. The remaining 14 TCCs were excluded from this test as either they were found to be reactive to the peptide mix representing the known epitopes upon retesting or they lost their proliferative potential (table S1). Of the 42 TCCs, 10 were reactive to at least one of the tested peptides (Fig. 2A). Of the 10 reactive TCCs, 3 were reactive to a single peptide, 4 were cross-reactive to two peptides, and the remaining 3 TCCs were cross-reactive to three peptides (Fig. 2A). The observed cross-reactivity was likely due to a high sequence similarity between the stimulatory peptides. We then tested these seven cross-reactive TCCs with titrated concentrations of the stimulatory peptides (Fig. 2B). Differential reactivities were observed, and this allowed us to identify one peptide being chiefly stimulatory for each TCC. Together, we identified five gliadin/glutenin peptide sequences representing the potential novel gliadin/glutenin epitopes (P2359, P2361, P2362, P2366, and P2369) that stimulated gliadin/glutenin-reactive TCCs unreactive to any hitherto identified gliadin/glutenin epitope (Fig. 2B).

To identify the true 9-nucleotide oligomer core sequences of these candidate epitopes, we designed shorter peptides derived from the 13-nucleotide oligomer peptides representing all the possible 9-nucleotide oligomer HLA-DQ2.5 peptide–binding registers (Fig. 2C). All of these candidate 9-nucleotide oligomer sequences were flanked by two glycine residues at the N-terminal end and one glycine residue at the C-terminal end. By performing T cell proliferation assays with 10 TCCs, we identified 5 TCCs that were reactive to the tested candidate peptides, of which 2 TCCs were cross-reactive (Fig. 2C and Table 2). Although the remaining five TCCs showed proliferative response to the originally tested 13-nucleotide oligomer peptides representing predicted epitopes, they failed to respond to the shorter peptides that represented each of the possible peptide-binding registers (fig. S2). Therefore, we were unable to identify the 9-nucleotide oligomer core region stimulating these TCCs. We surmise that these TCCs also recognize residues outside of the 9-nucleotide oligomer core region.

**Table 2.** List of identified gliadin/glutenin epitopes. Column 1 shows the names of TCCs reactive to shorter peptide sequences representing all possible peptide binding registers of the 13-nucleotide oligomer peptides. Column 2 shows stimulatory 13-nucleotide oligomer peptide sequences and their peptide IDs. The potential TG2 deamidation sites are underlined in the sequences. Column 3 provides the sequences of the identified 9-nucleotide oligomer peptide–binding registers (epitope sequences). The protein origins of the stimulatory peptides are displayed in column 4. Column 5 provides tentative names assigned to the identified epitopes.

TCC name/s	Stimulatory 13-nucleotide oligomer peptide (peptide ID)	Peptide binding register	Origin	Tentative epitope name
2135.2.10	LQPFPQPQQPYPQ (P2361)	PQPEQPYPQ	α-Gliadin	DQ2.5-glia-α4
5037.2.36	QQPFPQQPYPQQP (P2362)	PFPEQPYPE	ω-Gliadin	DQ2.5-glia-ω3
2135.2.37	PYPQPQPQYPQPQ (P2366)	PQPEYPQPQ	α-Gliadin	DQ2.5-glia-α5
2126.2.1	PFPQPQQQLPQPQ (P2369)	PFPQPEQEL	γ-Gliadin	DQ2.5-glia-γ6
2126.2.39		PQPEQELPQ		DQ2.5-glia-γ4f

Together, we were able to identify five previously unknown epitopes, of which two originated from α-gliadin, two from γ-gliadin, and one from ω-gliadin. On the basis of previously established nomenclature, the tentative names DQ2.5-glia-α4 (PQPEQPYPQ), DQ2.5-glia-α5 (PQPEYPQPQ), DQ2.5-glia-γ4f (PFPQPEQEL), DQ2.5-glia-γ6 (PFPQPEQEL), and DQ2.5-glia-ω3 (PFPEQPYPE) are suggested (Table 2). When we mapped these five epitopes back to the tested 13-nucleotide oligomers, three of them matched with the top original 9-nucleotide oligomers that were predicted by the two strategies (DQ2.5-glia-α4, DQ2.5-glia-γ4f, and DQ2.5-glia-ω3). Owing to the high sequence similarity of the gluten peptides, the remaining two epitopes (DQ2.5-glia-α5 and DQ2.5-glia-γ6) were serendipitously identified as we were testing 13-nucleotide oligomer sequences in the original screening. Although DQ2.5-glia-α5 was present in the list of 9-nucleotide oligomer sequences generated by strategy 2 (A and B), it did not score high enough to be tested in our study, suggesting that predicted 9-nucleotide oligomer peptides throughout the list are good candidates for gliadin/glutenin epitopes. The peptide sequence of DQ2.5-glia-γ6 was absent in the list; however, a very similar sequence with only one amino acid difference at P9 (L➔F) was predicted by strategy 2 (A and B).

## DISCUSSION

CD4^+^ T cells recognizing peptides from cereal gluten proteins of wheat, barley, and rye are the key players in the pathogenesis of CeD (*24*). Extensive previous work has identified 27 disease-driving gluten T cell epitopes (*11*). Still, many epitopes remain to be identified (*13*, *14*). While the identification of disease-driving gluten peptide sequences has transformed our current understanding of CeD, the full use of this insight in the food industry and for the clinical benefit of patients will require complete knowledge of gluten peptides that elicit CD4^+^ T cell activation in CeD. Novel and more efficient strategies are needed to identify these T cell epitopes.

Here, we aimed to develop reproducible, scaleable, and efficient knowledge-based strategies to identify CeD-relevant T cell epitopes. To this end, we generated TCCs and used T cell proliferation as a readout, which is a well-established method for ascertainment of T cell reactivity (*25*). As identification of any epitope is based on the availability of unique TCCs, an efficient way to obtain relevant T cells is critical. In previous studies, TCCs with unknown reactivity were mostly generated by direct cloning (*13*, *14*, *26*). However, such approach is laborious and inefficient for generating TCCs reactive to less prevalent gluten epitopes (*13*). Therefore, we have used TCCs generated from gut-derived T cells expressing phenotypic cell-surface markers that are hallmarks of gluten-reactive T cells. This allowed us to enrich for CeD-relevant T cells. Further, to increase the chances of selecting T cells with unknown epitope specificity, we also used a mixture of HLA-DQ:gluten tetramers to exclude T cells reactive to known immunodominant epitopes (*22*, *23*). Using this approach, we were able to generate phenotypically distinct gliadin/glutenin-reactive TCCs with unknown epitope specificity that served as ideal tools for testing the candidate peptides for gliadin/glutenin epitope discovery.

For gluten epitope identification, in addition to the requirement of unique TCCs as discussed in Sollid *et al.* (*2*), it is mandatory to identify the 9-nucleotide oligomer core region of the epitope involved in HLA-DQ binding to ascertain its correct binding register. Frequently, gluten T cell epitopes are concatemerized, as exemplified by the six overlapping epitopes within the well-known 33-nucleotide oligomer fragment of α-gliadin (*27*, *28*). Thus, with the feature that several 9-nucleotide oligomer core regions overlap each other in longer sequences, identifying the true epitope 9-nucleotide oligomer core region can be a challenge.

To identify the candidate peptide sequences for epitope discovery, we exploited features of the hitherto identified gliadin/glutenin epitopes as they contain imprints of the stringent T cell epitope selection in CeD. In the first strategy, we used the motif representing the residues in the four immunodominant gliadin/glutenin epitopes as bait and were able to identify 12 candidates for previously uncharacterized gliadin/glutenin epitopes in the database. Although this approach was successful in identifying gliadin/glutenin epitopes, it was not scalable beyond these 12 candidate peptides. On the other hand, the second strategy, based on several key features of the known gliadin/glutenin epitopes, resulted in the identification of hundreds of potential candidate peptide sequences. Here, we only tested a fraction of these peptides for T cell recognition; hence, hundreds more sequences remain to be tested. The 9-nucleotide oligomer peptide binding register of several candidate epitopes identified by our strategies lie within previously reported 20-nucleotide oligomer peptides identified using peripheral blood T cells (*12*). These 20-nucleotide oligomer peptides were demonstrated to contribute to the T cell recall response to gluten in HLA-DQ2.5 patients. Here, despite only testing a limited number of candidate peptide sequences, we were able to identify five previously unknown epitopes with defined 9-nucleotide oligomer core regions. We tentatively name the epitopes DQ2.5-glia-α4 (PQPEQPYPQ), DQ2.5-glia-α5 (PQPEYPQPQ), DQ2.5-glia-γ4f (PQPEQELPQ), DQ2.5-glia-γ6 (PFPQPEQEL), and DQ2.5-glia-ω3 (PFPEQPYPE). From the five identified gliadin/glutenin epitopes, three matched the originally identified top scoring 9-nucleotide oligomers selected for testing. The other two epitopes were concatemerized with the epitopes we predicted, possibly reflecting the known feature of overlapping gluten T cell epitopes.

While our strategy proved successful in identifying previously uncharacterized epitopes, we only assigned reactivity to 10 of 42 TCCs. To identify the epitopes recognized by the remaining TCC, testing more extensive panel of peptides would be required. Another limitation of our study is that we have considered only the features of hitherto known HLA-DQ2.5–restricted gliadin/glutenin epitopes for identifying candidate epitopes. The unknown gliadin/glutenin epitopes that do not strictly follow these set of parameters or are restricted by another HLA-DQ haplotype will likely not be identified by the approach presented here. In addition, our study was biased toward discovery of novel epitopes from wheat gliadin/glutenin. To tackle the latter two mentioned limitations, the strategy, and hence the algorithm, could be modified to consider known epitopes from other grains, such as barley and rye, and epitopes restricted by other CeD-related haplotypes, mainly HLA-DQ2.2 and HLA-DQ8. An untargeted approach testing large panels of synthetic gluten peptides, as previously reported (*12*), would likely identify additional CeD-driving epitopes. Unlike the targeted approach used here, the comprehensive untargeted approach is substantially more resource and labor demanding.

We demonstrated that by generating TCCs with phenotype distinct to the gluten-reactive T cells and by harnessing sequence signatures of known gliadin/glutenin epitopes, we can successfully identify gliadin/glutenin epitopes driving CeD. To complete a quest for the identification of all CeD-relevant T cell epitopes, a more exhaustive study would have to be performed by testing more candidate peptides against a larger number of TCCs. Discovering additional gluten epitopes will have substantial food safety and clinical implications.

## MATERIALS AND METHODS

### Study design

The main aim of this study was to develop an efficient method for identification of yet unidentified gluten epitopes. To do that, we obtained gluten-reactive TCCs with unknown epitope specificity. This was done by assessing the grain reactivity and epitope specificity of TCCs isolated on the basis of the phenotype typical for gluten-reactive T cells. To increase the chances of obtaining TCCs with unknown gluten epitope specificity, we only selected for TCCs that previously did not bind to HLA-DQ2.5 tetramers representing five immunodominant gluten epitopes. In addition, we designed an efficient strategy to identify candidate gluten epitopes. This was done by using the prior knowledge of the hitherto identified gluten epitopes to generate candidate peptide sequences and subsequently searching the GluProI *T. aestivum* database for their presence. To identify the stimulatory candidate gluten epitopes, gluten-reactive TCCs with unknown epitope specificity were tested against a panel of peptides generated using the knowledge-based strategies. All experiments were performed at least twice (unless stated otherwise) in triplicate, and the results show one representative independent experiment.

### Study approval

The study was approved by the Regional Committee for Medical and Health Research Ethics South-East Norway (project no. 6544). All patient samples were collected with an informed written consent from patients who visited the endoscopy unit at Oslo University Hospital—Rikshospitalet or the endoscopy unit at Akershus University Hospital for the endoscopic routine procedure for the diagnosis of CeD.

### Generation of TCCs

The TCCs used in the current study were generated from biopsy samples from five untreated patients with CeD (CD2126, CD2135, CD5030, CD5037, and CD5063) recruited for a different study (*22*). All these patients carried the HLA-DQ2.5 haplotype but not the HLA-DQ2.2 or the HLA-DQ8 haplotypes. Single T cells were sorted on the basis of the previously described phenotype (CD3^+^, CD4^+^, PD-1^+^, CD39^+^, CD161^+^, ICOS^+^, CXCR3^+^, CD25^−^, and CD127-) (*22*, *23*). These T cells were further sorted in the antigen-free culture medium as two subpopulations depending on whether they stained with HLA-DQ2.5:gliadin/glutenin tetramers (Ttet^+^) or not (Ttet-). The antigen-free culture medium was composed of feeder mix {irradiated [60 gray (Gy)] peripheral mononuclear cells from three healthy donors, 1 × 10^6^/ml} in 10% human serum (HS) , RPMI 1640 (Sigma-Aldrich), supplemented with penicillin-streptomycin (Sigma-Aldrich), 0.01 M β-mercapthoethanol (Sigma-Aldrich), interleukin-2 (IL-2, 10 IU/ml; Amersham Pharmacia Biotech AB), IL-15 (1 ng/ml; R&D Systems), and phytohemagglutinin (PHA) (1 μg/ml; Murex). TCCs were generated by performing cloning by limiting dilution (one TCC per three wells in a volume of 20 μl) using Terasaki plates (VWR). TCCs were cultured for 10 days in Terasaki plates that were placed in a moist chamber inside a humidified incubator at 37°C with 5% CO_2._ All growing TCCs were expanded for an additional 10 days into a well of a 48-well culture plate (Corning) with antigen-free culture medium described above. The TCCs were supplemented with additional medium (10% HS, RPMI 1640, Sigma-Aldrich), supplemented with penicillin-streptomycin (Sigma-Aldrich), 0.01 M β-mercapthoethanol (Sigma-Aldrich), IL-2 (10 IU/ml; Amersham Pharmacia Biotech AB), and IL-15 (1 ng/ml; R&D Systems) as required during the 10 days. The TCCs were either used directly from culture or cryopreserved before being used in T cell proliferation assays.

### Extraction and deamidation of gliadin/glutenin, hordein, and secalin

Wheat (gliadin/gluteninin), barley (hordein), and rye (secalin) proteins were extracted in-house from grocery store flours, wheat (Møllerens—siktet hvetemel), barley (Regal—byggmel), and rye (Møllerens—siktet rugmel), as described previously (*29*). Briefly, gliadins/glutenins, hordeins, and secalins were extracted from defatted flour using 0.5 M tris-HCl (pH 6.8) with 50% propanol and 2% dithiothreitol. After 1 hour of incubation at 65°C, samples were precipitated using 1.5% NaCl and dissolved in 0.01 M NH_4_HCO_3_ also containing 2 M urea. The proteins were digested with chymotrypsin (Sigma-Aldrich) at 1:100 w/w at 37°C for 5 hours and later dialyzed twice against NH_4_HCO_3_ (cutoff, 1 kDa), at first for 5 hours at 4°C and later overnight also at 4°C. Dialyzed proteins were stored as freeze-dried pellets at −20°C. The proteins were then resuspended in 100 mM (pH 7.4) tris-HCl containing 2 mM CaCl_2_ for a protein concentration of 1 mg/ml and deamidated using in-house–produced recombinant human TG2 (50 μg/ml) for 2 hours at 37°C. The deamidated proteins were used in T cell proliferation assays.

### Synthesis and deamidation of peptides

All the peptides used in this study were commercially synthesized by GenScript at a minimal purity of 90% and with quality assessment by reversed-phase high-performance liquid chromatography and electrospray ionization mass spectrometry. No additional modifications were introduced into synthesized peptides with the exception of peptides with N-terminal glutamine (Q) that were N-terminally acetylated to prevent pyroglutamate formation. Peptides in Table 2 and table S3 were synthesized in their deamidated state, while peptides in Table 1 were synthesized in their nondeamidated state. The peptides in Table 1 were deamidated by TG2 before being used in the T cell proliferation assay. The peptides were resuspended in 100 mM (pH 7.4) tris-HCl containing 2 mM CaCl_2_ for a protein concentration of 500 μM in a volume of 500 μl and deamidated using in-house–produced TG2 (100 μg/ml) for 2 hours at 37°C.

### T cell proliferation assay

T cell proliferation assay was performed as described earlier (*30*). As antigen-presenting cells (APCs), we used immortalized B cell lines expressing HLA-DQ2.5, either Raji Burkitt’s lymphoma cell line (CCL-86) or an Epstein-Barr virus–transformed cell line of an HLA-DQ2.5 homozygous CeD patient (CD114). The measurements were performed in either duplicate or triplicate. On day 1, 75,000 irradiated APCs (75 Gy) at a volume of 75 μl were added to wells containing 25 μl of antigen (experiment-defined concentration) and incubated overnight at 37°C. The following day, 50,000 TCCs at a volume of 50 μl were added to the respective wells. On day 3 (for TCCs in culture) or day 4 (for frozen TCCs), 1 μCi ^3^H-thymidine (Hartman Analytics) diluted in 0.9% NaCl solution (Braun) was added to the wells. The ^3^H-thymidine incorporation was measured no later than 16 hours after ^3^H-thymidine addition to the plates. Cells were harvested using an automated harvester (Mach III, TomTec), and the thymidine incorporation was measured by liquid stimulation counting (Wallac MicroBeta TriLux 1450, PerkinElmer) as counts per minute (CPM). The stimulation of the TCCs by the tested antigen was assessed using the stimulation index (SI) calculated using the following formula

$$\begin{array}{rl} & \mathrm{SI}=\mathrm{mean}\;\mathrm{CPM}\;\mathrm{for}\;a\;\mathrm{given}\;\mathrm{antigen}/\mathrm{mean}\;\mathrm{CPM}\;\mathrm{for}\;\mathrm{the}\;\mathrm{negative}\\ & \mathrm{control}\;(\mathrm{solvent}) \end{array}$$

TCCs were considered reactive when the SI was greater than or equal to 1.8 and 3 using Raji cells and CD114 cells, respectively.

### Computational model for generating candidate gluten epitopes

The “Advanced algorithm database search” (strategy 2), as described in Results, was implemented in Python (version 3.8). The code reads in a training set of known gluten epitopes, estimates a mixture model of sequence preference according to alternative deamidation patterns, and outputs predicted gluten epitopes according to this model. The output is given in deamidated and nondeamidated versions and with or without filtering and sorting of candidate epitopes according to their presence in provided fasta files (GluProI database) of gluten proteins.

### Data analysis and statistics

Statistical analysis was performed using GraphPad Prism software (version 8) (San Diego). Flow cytometry data were analyzed using FlowJo software (Three Star). All the figures were generated in Adobe Illustrator.

## Notes

View/request a protocol for this paper from *Bio-protocol*.
