# Supplementary material for: Identification of gluten T cell epitopes driving celiac disease
Source: Sci Adv. 2023 Jan 25;9(4):eade5800. doi: 10.1126/sciadv.ade5800 (PMC9876541; doi:10.1126/sciadv.ade5800)
Supplement: Supplementary file 1 — Figs. S1 and S2 Tables S1 to S3 [file sciadv.ade5800_sm.pdf]

Science Advances
AAAS

Supplementary Materials for

Identification of gluten T cell epitopes driving celiac disease

Markéta Chlubnová *et al.*

Corresponding author: Markéta Chlubnová, marketch@medisin.uio.no;
Shiva Dahal-Koirala, shiva.dahal-koirala@medisin.uio.no; Ludvig M. Sollid, l.m.sollid@medisin.uio.no

Sci. Adv. **9**, eade5800 (2023)
DOI: 10.1126/sciadv.ade5800

This PDF file includes:

Figs. S1 and S2
Tables S1 to S3

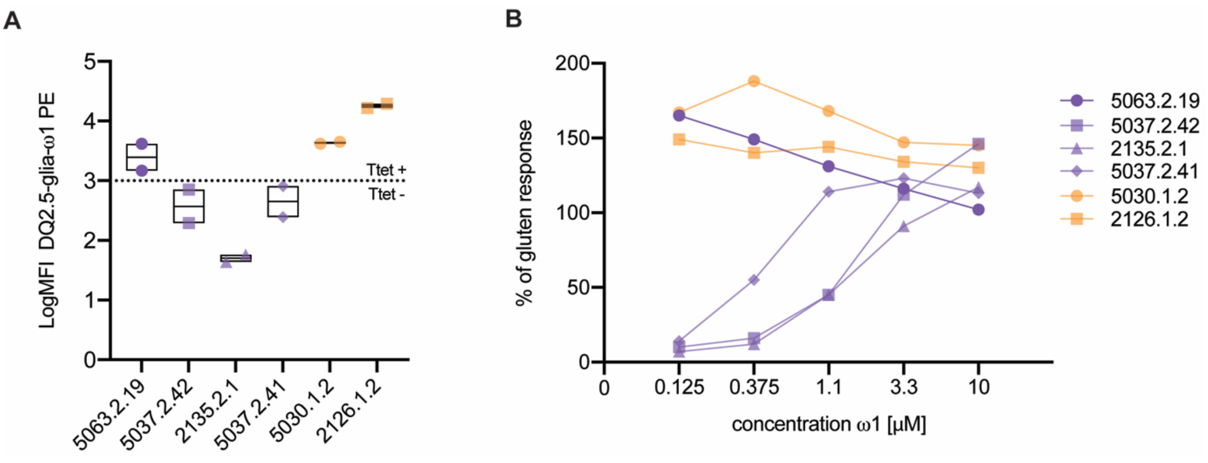

Figure S1. Correlation between staining intensity and peptide concentration necessary to induce proliferative response of TCCs. A) We selected five TCCs that were previously reactive to peptide representing DQ2.5-glia-ω1 epitope (10μM) in T-cell proliferation assay. We stained these TCCs with PE-labeled HLA-DQ2.5-glia-ω1 tetramers and determined LogMFI values for each TCC. TCCs with LogMFI value < 3 were considered tetramer negative (Ttet-), whereas TCCs with LogMFI > 3 were considered tetramer positive (Ttet+). Two data points for each TCC represent two individual experiments. B) We measured a proliferative response of each TCC to titrated (range 0.125μM-10μM) peptide representing DQ2.5-glia-ω1 epitope in a T-cell proliferation assay. The results are displayed as proliferative response of each TCC relative to their response to gluten. This experiment was performed twice, and the representative results is displayed here. *Violet coloring represents Ttet- TCCs and orange coloring represents Ttet+ TCCs. All five tested TCCs were expressing the phenotype characteristic of gluten-specific T cells.*

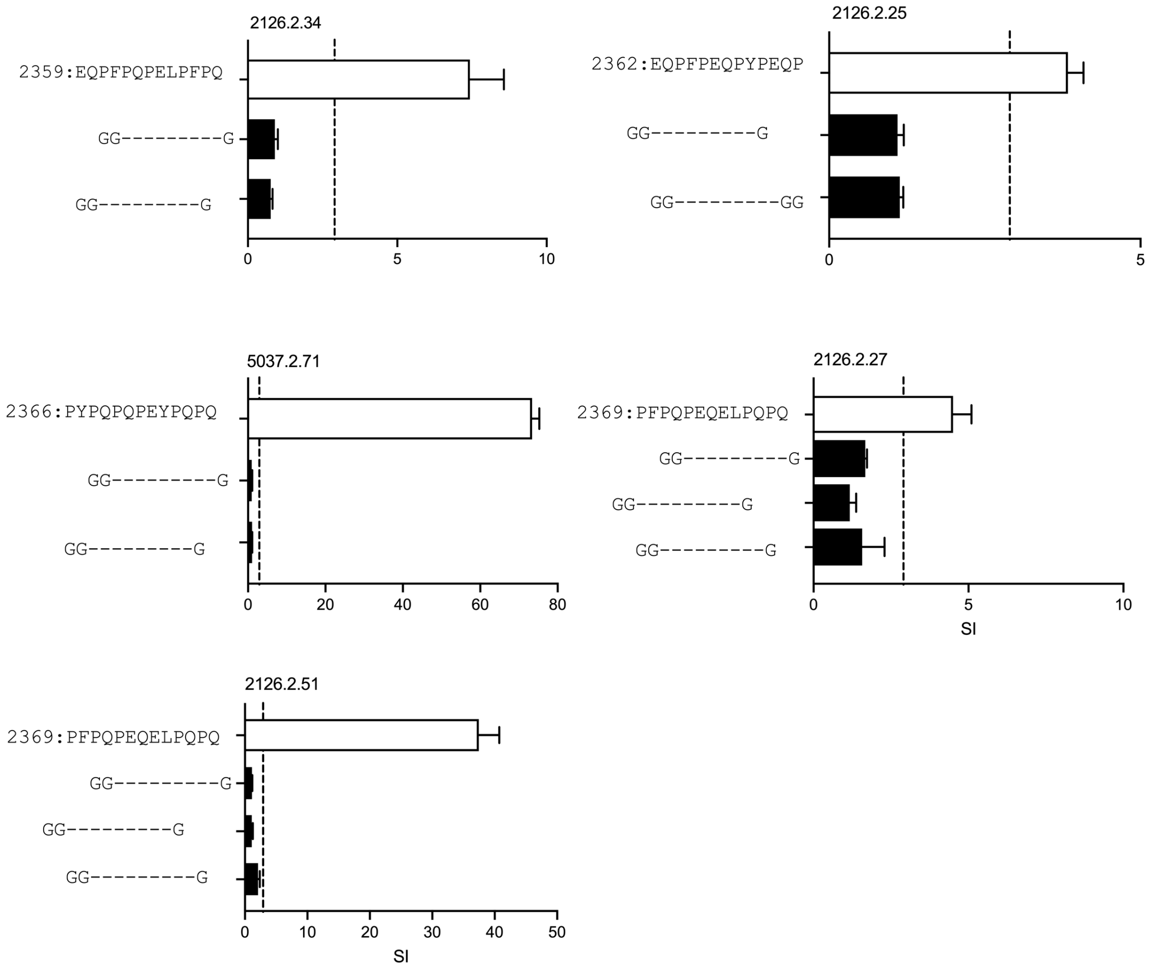

Figure S2. Reactivity of the remaining five TCCs (from Figure 2). TCCs with previously defined stimulatory 13-mer peptide sequence were tested against shorter peptides representing respective possible peptide binding register variants flanked with two glycine residues at N-terminus and one glycine residue at the C-terminus in a T-cell proliferation assay. White bars represent original stimulatory 13-mer sequences for each TCC, whereas black bars represent the reactivity to shorter peptides containing only a single possible candidate epitope sequence. TCCs that were not reactive to the corresponding shorter peptides are displayed. Error bars represent standard deviation. All peptides were tested at 10μM concentration in triplicates. The TCCs were considered reactive if SI > 3 (stippled line). Results from one representative experiment of two are shown.

Table S1. List of tested TCCs. List of all TCCs used in this study with indication of testing performed for each TCC and its outcome.

Table S2. Peptide sequences of 20 known unique HLA-DQ2.5 restricted T-cell epitopes in celiac disease. Column two shows the in house assigned peptide ID. Column three and four enlists the name of the epitope and the respective 9-mer peptide binding register. The last column shows the peptide sequence representing the epitope where the 9-mer core sequence of the epitope is bold faced.

Table S3. List of peptides generated by strategy 2A and 2B. Top 100 candidate peptides generated by each strategy.

Table S1

TCC name	Grains (n=3)		Known epitopes (n=20)		Potential novel gluten epitopes (n=18)		Shorter peptides (n=11)		Comment
	Tested	Reactive	Tested	Reactive	Tested	Reactive	Tested	Reactive	
2126.2.12	yes	no	yes	no	no	n/a	no	n/a	-
2126.2.13	yes	no	yes	no	no	n/a	no	n/a	-
2126.2.14	yes	no	yes	no	no	n/a	no	n/a	-
2126.2.16	yes	no	yes	no	no	n/a	no	n/a	-
2126.2.17	yes	no	yes	no	no	n/a	no	n/a	-
2126.2.18	yes	no	yes	no	no	n/a	no	n/a	-
2126.2.19	yes	no	yes	no	no	n/a	no	n/a	-
2126.2.2	yes	no	yes	no	no	n/a	no	n/a	-
2126.2.20	yes	no	yes	no	no	n/a	no	n/a	-
2126.2.21	yes	no	yes	no	no	n/a	no	n/a	-
2126.2.23	yes	no	yes	no	no	n/a	no	n/a	-
2126.2.24	yes	no	yes	no	no	n/a	no	n/a	-
2126.2.30	yes	no	yes	no	no	n/a	no	n/a	-
2126.2.32	yes	no	yes	no	no	n/a	no	n/a	-
2126.2.33	yes	no	yes	no	no	n/a	no	n/a	-
2126.2.35	yes	no	yes	no	no	n/a	no	n/a	-
2126.2.36	yes	no	yes	no	no	n/a	no	n/a	-
2126.2.37	yes	no	yes	no	no	n/a	no	n/a	-
2126.2.38	yes	no	yes	no	no	n/a	no	n/a	-
2126.2.4	yes	no	yes	no	no	n/a	no	n/a	-
2126.2.40	yes	no	yes	no	no	n/a	no	n/a	-
2126.2.41	yes	no	yes	no	no	n/a	no	n/a	-
2126.2.42	yes	no	yes	no	no	n/a	no	n/a	-
2126.2.43	yes	no	yes	no	no	n/a	no	n/a	-
2126.2.45	yes	no	yes	no	no	n/a	no	n/a	-
2126.2.46	yes	no	yes	no	no	n/a	no	n/a	-
2126.2.47	yes	no	yes	no	no	n/a	no	n/a	-
2126.2.5	yes	no	yes	no	no	n/a	no	n/a	-
2126.2.50	yes	no	yes	no	no	n/a	no	n/a	-
2126.2.52	yes	no	yes	no	no	n/a	no	n/a	-
2126.2.53	yes	no	yes	no	no	n/a	no	n/a	-
2126.2.6	yes	no	yes	no	no	n/a	no	n/a	-
2126.2.7	yes	no	yes	no	no	n/a	no	n/a	-
2126.2.8	yes	no	yes	no	no	n/a	no	n/a	-
2135.2.18	yes	no	yes	no	no	n/a	no	n/a	-

2135.2.28	yes	no	yes	no	no	n/a	no	n/a	-
2135.2.30	yes	no	yes	no	no	n/a	no	n/a	-
2135.2.32	yes	no	yes	no	no	n/a	no	n/a	-
2135.2.33	yes	no	yes	no	no	n/a	no	n/a	-
2135.2.42	yes	no	yes	no	no	n/a	no	n/a	-
2135.2.5	yes	no	yes	no	no	n/a	no	n/a	-
2135.2.6	yes	no	yes	no	no	n/a	no	n/a	-
2135.2.8	yes	no	yes	no	no	n/a	no	n/a	-
5030.2.2	yes	no	yes	no	no	n/a	no	n/a	-
5037.2.1	yes	no	yes	no	no	n/a	no	n/a	-
5037.2.12	yes	no	yes	no	no	n/a	no	n/a	-
5037.2.13	yes	no	yes	no	no	n/a	no	n/a	-
5037.2.17	yes	no	yes	no	no	n/a	no	n/a	-
5037.2.21	yes	no	yes	no	no	n/a	no	n/a	-
5037.2.25	yes	no	yes	no	no	n/a	no	n/a	-
5037.2.26	yes	no	yes	no	no	n/a	no	n/a	-
5037.2.28	yes	no	yes	no	no	n/a	no	n/a	-
5037.2.29	yes	no	yes	no	no	n/a	no	n/a	-
5037.2.34	yes	no	yes	no	no	n/a	no	n/a	-
5037.2.38	yes	no	yes	no	no	n/a	no	n/a	-
5037.2.4	yes	no	yes	no	no	n/a	no	n/a	-
5037.2.48	yes	no	yes	no	no	n/a	no	n/a	-
5037.2.51	yes	no	yes	no	no	n/a	no	n/a	-
5037.2.58	yes	no	yes	no	no	n/a	no	n/a	-
5037.2.61	yes	no	yes	no	no	n/a	no	n/a	-
5037.2.65	yes	no	yes	no	no	n/a	no	n/a	-
5037.2.67	yes	no	yes	no	no	n/a	no	n/a	-
5037.2.69	yes	no	yes	no	no	n/a	no	n/a	-
5037.2.70	yes	no	yes	no	no	n/a	no	n/a	-
5063.2.1	yes	no	yes	no	no	n/a	no	n/a	-
5063.2.12	yes	no	yes	no	no	n/a	no	n/a	-
5063.2.13	yes	no	yes	no	no	n/a	no	n/a	-
5063.2.15	yes	no	yes	no	no	n/a	no	n/a	-
5063.2.16	yes	no	yes	no	no	n/a	no	n/a	-
5063.2.18	yes	no	yes	no	no	n/a	no	n/a	-
5063.2.2	yes	no	yes	no	no	n/a	no	n/a	-
5063.2.21	yes	no	yes	no	no	n/a	no	n/a	-
5063.2.22	yes	no	yes	no	no	n/a	no	n/a	-
5063.2.32	yes	no	yes	no	no	n/a	no	n/a	-
5063.2.9	yes	no	yes	no	no	n/a	no	n/a	-
2135.2.24	yes	no	yes	no	no	n/a	no	n/a	-
2135.2.1	yes	yes	yes	yes	no	n/a	no	n/a	-
5037.2.41	yes	yes	yes	yes	no	n/a	no	n/a	-
5037.2.66	yes	yes	yes	yes	no	n/a	no	n/a	-
5037.2.14	yes	yes	yes	yes	no	n/a	no	n/a	-
5037.2.55	yes	yes	yes	yes	no	n/a	no	n/a	-
5063.2.5	yes	yes	yes	yes	no	n/a	no	n/a	-
2135.2.13	yes	yes	yes	yes	no	n/a	no	n/a	-

5037.2.11	yes	yes	yes	yes	no	n/a	no	n/a	-
5037.2.16	yes	yes	yes	yes	no	n/a	no	n/a	-
5037.2.2	yes	yes	yes	yes	no	n/a	no	n/a	-
5037.2.42	yes	yes	yes	yes	no	n/a	no	n/a	-
5037.2.47	yes	yes	yes	yes	no	n/a	no	n/a	-
5037.2.52	yes	yes	yes	yes	no	n/a	no	n/a	-
5037.2.6	yes	yes	yes	yes	no	n/a	no	n/a	-
5063.2.11	yes	yes	yes	yes	no	n/a	no	n/a	-
5063.2.17	yes	yes	yes	yes	no	n/a	no	n/a	-
5063.2.19	yes	yes	yes	yes	no	n/a	no	n/a	-
5063.2.23	yes	yes	yes	yes	no	n/a	no	n/a	-
5063.2.6	yes	yes	yes	yes	no	n/a	no	n/a	-
2126.2.11	yes	yes	yes	yes	no	n/a	no	n/a	-
2126.2.28	yes	yes	yes	yes	no	n/a	no	n/a	-
2126.2.44	yes	yes	yes	yes	no	n/a	no	n/a	-
2126.2.49	yes	yes	yes	yes	no	n/a	no	n/a	-
2126.2.9	yes	yes	yes	yes	no	n/a	no	n/a	-
2135.2.15	yes	yes	yes	yes	no	n/a	no	n/a	-
2135.2.36	yes	yes	yes	yes	no	n/a	no	n/a	-
2135.2.9	yes	yes	yes	yes	no	n/a	no	n/a	-
5030.2.1	yes	yes	yes	yes	no	n/a	no	n/a	-
5030.2.5	yes	yes	yes	yes	no	n/a	no	n/a	-
5037.2.19	yes	yes	yes	yes	no	n/a	no	n/a	-
5037.2.22	yes	yes	yes	yes	no	n/a	no	n/a	-
5037.2.33	yes	yes	yes	yes	no	n/a	no	n/a	-
5037.2.39	yes	yes	yes	yes	no	n/a	no	n/a	-
5037.2.44	yes	yes	yes	yes	no	n/a	no	n/a	-
5037.2.5	yes	yes	yes	yes	no	n/a	no	n/a	-
5037.2.50	yes	yes	yes	yes	no	n/a	no	n/a	-
5037.2.59	yes	yes	yes	yes	no	n/a	no	n/a	-
5037.2.64	yes	yes	yes	yes	no	n/a	no	n/a	-
5037.2.9	yes	yes	yes	yes	no	n/a	no	n/a	-
5063.2.14	yes	yes	yes	yes	no	n/a	no	n/a	-
5063.2.26	yes	yes	yes	yes	no	n/a	no	n/a	-
5063.2.33	yes	yes	yes	yes	no	n/a	no	n/a	-
5030.2.7	yes	yes	yes	yes	no	n/a	no	n/a	-
5037.2.18	yes	yes	yes	yes	no	n/a	no	n/a	-
5037.2.20	yes	yes	yes	yes	no	n/a	no	n/a	-
5037.2.23	yes	yes	yes	yes	no	n/a	no	n/a	-
5037.2.63	yes	yes	yes	yes	no	n/a	no	n/a	-
2126.2.26	yes	yes	yes	yes	no	n/a	no	n/a	reactive to epitope mix
2126.2.29	yes	yes	yes	yes	no	n/a	no	n/a	reactive to epitope mix
2126.2.31	yes	yes	yes	yes	no	n/a	no	n/a	reactive to epitope mix
2126.2.48	yes	yes	yes	yes	no	n/a	no	n/a	reactive to epitope mix
2135.2.22	yes	yes	yes	yes	no	n/a	no	n/a	reactive to epitope mix
2135.2.34	yes	yes	yes	yes	no	n/a	no	n/a	reactive to epitope mix
5037.2.15	yes	yes	yes	yes	no	n/a	no	n/a	reactive to epitope mix
5037.2.40	yes	yes	yes	yes	no	n/a	no	n/a	reactive to epitope mix

2126.2.15	yes	yes	yes	no	no	n/a	no	n/a	lost reactivity to gluten
2126.2.22	yes	yes	yes	no	no	n/a	no	n/a	lost reactivity to gluten
2135.2.14	yes	yes	yes	no	no	n/a	no	n/a	lost reactivity to gluten
5037.2.32	yes	yes	yes	no	no	n/a	no	n/a	lost reactivity to gluten
5037.2.56	yes	yes	yes	no	no	n/a	no	n/a	lost reactivity to gluten
5063.2.24	yes	yes	yes	no	no	n/a	no	n/a	lost reactivity to gluten
5063.2.10	yes	yes	yes	no	yes	no	no	n/a	-
2135.2.10	yes	yes	yes	no	yes	yes	yes	yes	P2361 (P2362, P2366)/2382
2126.2.1	yes	yes	yes	no	yes	yes	yes	yes	P2369 (P2371, P2373)/2377
2126.2.10	yes	yes	yes	no	yes	no	no	n/a	-
2126.2.25	yes	yes	yes	no	yes	yes	yes	no	P2362 (P2372, P2373)
2126.2.27	yes	yes	yes	no	yes	yes	yes	no	P2369
2126.2.34	yes	yes	yes	no	yes	yes	yes	no	P2359 (P2368)
2126.2.39	yes	yes	yes	no	yes	yes	yes	yes	P2369 (P2371, P2373)/P2377
2126.2.51	yes	yes	yes	no	yes	yes	yes	no	P2369 (P2375)
2126.2.54	yes	yes	yes	no	yes	no	no	n/a	-
2135.2.11	yes	yes	yes	no	yes	no	no	n/a	-
2135.2.16	yes	yes	yes	no	yes	no	no	n/a	-
2135.2.19	yes	yes	yes	no	yes	no	no	n/a	-
2135.2.21	yes	yes	yes	no	yes	no	no	n/a	-
2135.2.26	yes	yes	yes	no	yes	no	no	n/a	-
2135.2.27	yes	yes	yes	no	yes	no	no	n/a	-
2135.2.3	yes	yes	yes	no	yes	no	no	n/a	-
2135.2.35	yes	yes	yes	no	yes	no	no	n/a	-
2135.2.37	yes	yes	yes	no	yes	yes	yes	yes	P2366/P2380
2135.2.38	yes	yes	yes	no	yes	no	no	n/a	-
2135.2.4	yes	yes	yes	no	yes	no	no	n/a	-
2135.2.44	yes	yes	yes	no	yes	no	no	n/a	-
2135.2.45	yes	yes	yes	no	yes	no	no	n/a	-
5030.2.4	yes	yes	yes	no	yes	no	no	n/a	-
5030.2.6	yes	yes	yes	no	yes	no	no	n/a	-
5037.2.24	yes	yes	yes	no	yes	no	no	n/a	-
5037.2.3	yes	yes	yes	no	yes	no	no	n/a	-
5037.2.30	yes	yes	yes	no	yes	no	no	n/a	-
5037.2.31	yes	yes	yes	no	yes	no	no	n/a	-
5037.2.35	yes	yes	yes	no	yes	no	no	n/a	-
5037.2.36	yes	yes	yes	no	yes	yes	yes	yes	P2362/P2383
5037.2.43	yes	yes	yes	no	yes	no	no	n/a	-
5037.2.45	yes	yes	yes	no	yes	no	no	n/a	-
5037.2.46	yes	yes	yes	no	yes	no	no	n/a	-
5037.2.53	yes	yes	yes	no	yes	no	no	n/a	-
5037.2.54	yes	yes	yes	no	yes	no	no	n/a	-
5037.2.57	yes	yes	yes	no	yes	no	no	n/a	-
5037.2.60	yes	yes	yes	no	yes	no	no	n/a	-
5037.2.68	yes	yes	yes	no	yes	no	no	n/a	-
5037.2.7	yes	yes	yes	no	yes	no	no	n/a	-
5037.2.71	yes	yes	yes	no	yes	yes	yes	no	P2366 (P2364)
5063.2.7	yes	yes	yes	no	yes	no	no	n/a	-

n/a=not applicable

Peptide ID main stimulatory peptide

(Petide ID other stimulatory peptides)

/Peptide ID shorter stimulatory peptide

Table S2

Nr	Peptide ID	Epitopes name	9-mer peptide binding register	Peptide sequence
1	P1314	glia-α1b	PYPQPELPY	PQL**PYPQPELPY**
2	P2345	glia-α3	FRPEQPYPQ	PQP**FRPEQPYPQ**PQ
3	P1213	glia-γ1	PQQSFPEQQ	PEQ**PQQSFPEQE**RP
4	P1298	glia-γ2	IQPEQPAQL	GI**IQPEQPAQL**
5	P1584	glia-γ3	QQPEQPYPQ	FP**EQPEQPYPE**Q
6	P1396	glia-γ4a	SQPEQEFPQ	F**SQPEQEFPQ**PQ
7	P1935	glia-γ4b	PQPEQEFPQ	FP**QPEQEFPQ**PQ
8	P1653	glia-γ4c	QQPEQPFPQ	T**EQPEQPFPQ**P
9	P2350	glia-γ4d	PQPEQPFCQ	PF**PQPEQPFCE**QP
10	P2351	glia-γ4e	LQPEQPFPQ	**LQPEQPFPE**QP
11	P1571	glia-γ5	QQPFPEQPQ	P**EQPFPEQPE**Q
12	P1269	glia-α1a	PFPQPELPY	QLQ**PFPQPELPY**
13	P1313	glia-α2	PQPELPYPQ	**PQPELPYPQ**PQL
14	P2046	glia-ω1	PFPQPEQPF	PQQ**PFPQPEQPF**P
15	P2047	glia-ω2	PQPEQPFPW	F**PQPEQPFPW**QP
16	P2109	glut-L1	PFSEQEQPV	QQP**PFSEQEQPV**LPQ
17	P2341	glut-L2	FSQQQESPF	PP**FSEQQESPF**SQ
18	P2204	hor-3a	PIPEQPQPY	PEQ**PIPEQPQPY**PQQP
19	P2342	hor-3b	PYPEQPQPY	PEQ**PYPEQPQPY**PQ
20	P2343	sec-3	PFPEQPEQI	PQ**PFPEQPEQI**IPQ

Table S3

Nr	Strategy 2A, n=100		Strategy 2B, n=100	
	Sequence	Score	Sequence	Score
1	PQPQPQQPQ	1964	QQPQQPFPQ	154536
2	PQPQQPFPQ	1683	PQPQLPYPQ	89271
3	PQPQQQLPQ	1543	PFPQPQQPQ	15543
4	PQPQQPYPQ	1500	PFPQQPYPQ	11232
5	PFPQPQQPQ	1197	LQPQQPFPQ	7020
6	PQPQLPFPQ	1146	QQPQLPFPQ	6615
7	PQPQLPYPQ	981	PQPQQPYPQ	6000
8	PFPQQPYPQ	936	PQPQQPFPW	5913
9	PQPQQPFPW	657	QQPFPQQPQ	5143
10	PFPQPQQPY	526	PQPQLPFPQ	4584
11	PFPQPQQPF	506	QQPQQPYPQ	3726
12	PQPQQQFPQ	430	PFPQPQLPY	3540
13	PFPQPQLPF	347	QFPQPQQPQ	2736
14	PFPQPQLPY	292	PQPQQPFCQ	2704
15	QQPQQPFPQ	274	PFSQQQQPV	2040
16	PQPQQQLPQ	243	PQPQQPQQQ	1725
17	QQPQQPYPQ	207	PQPQQPFPQ	1683
18	PQPQQPFPL	175	PQPQQQFPQ	1632
19	PQPQQPFCQ	169	PQPQLPYSQ	1512
20	IQPQQQQPQ	164	PFPQPQQPF	1496
21	FQPQQPYPQ	163	FRPQQPYPQ	1470
22	QFPQPQQPQ	162	IQPQQPFPQ	1068
23	PQQQQPFPQ	157	PFPQPQQAQ	912
24	PQPQPQQPQ	157	PQQQQPFPQ	785
25	PFPQQQQPV	156	PQPQQTFPQ	775
26	QQPQLPFPQ	147	SQQQQPFPQ	630
27	SQPQQQQPQ	147	PQPQPQQPQ	628
28	PFPQQQQPL	142	VQPQQQQPQ	608
29	PQQQQQLPQ	137	FPPQQPYPQ	594
30	QFPQQQLPQ	136	WQPQQPFPQ	360
31	PQPQPQYPQ	122	PFPQSQQPQ	360
32	LQPQQPFPQ	108	PFPQQQQPL	336
33	PFPQPQQPQ	99	PQPFPQQPP	310
34	IQPQQPFPQ	89	QQPQQPFQQ	300
35	LQPQQPYPQ	82	PFPHPQQPQ	290
36	PQPQQPYSQ	75	QQPQQSFPQ	290
37	PRPQLPYPQ	74	PQPQPQYPQ	280
38	PFSQQQQPQ	70	PFPQQQQPI	264
39	PQPQQQFPQ	65	QQPIQQQPQ	264
40	PSPQPQQPF	62	NQPQQQQPQ	261
41	PYPQPQQPF	62	QQQQQPFPQ	260
42	PQPQQPFSQ	60	QQPQQTFPQ	260
43	QQPQQPFQQ	60	KQPQQPFPQ	252
44	PFPHPQQPQ	58	PFSQQQQPS	248
45	LQPQLPYPQ	56	PQPQPQYSQ	248
46	NQPQQQQPQ	55	PQQQQQLPQ	242

47	PFPPPQQPQ	52	QQPQQPFPL	242
48	PFPQPQQPI	51	QQQQQQQPL	237
49	PFPQQQQPI	48	QQPQQQLGQ	234
50	PFPQQQQPP	47	PFPQLQQPQ	231
51	VQPQQQQPQ	46	HQPQQPYPQ	230
52	MQPQQPFPQ	44	PFPQTQQPQ	228
53	PFPQPQQPF	44	QRPQQPFPQ	220
54	PLPQPQQPQ	43	QQPYPQQPQ	220
55	PFPQLQQPQ	41	QLPQPQQPQ	216
56	QQPQQQFPQ	40	QQPQQPFLQ	216
57	WQPQQPFPQ	40	LGQQPQQQQ	197
58	PQPQLPYLQ	39	PFSQQQQPQ	196
59	RQPQQPYPQ	39	QQPFPQQPY	195
60	PFPQSQQPQ	39	FPQQPQQPF	182
61	PFPQTQQPQ	38	PQPQQPQQP	179
62	QQPFPQQPQ	37	PFPQPQQPY	175
63	PFSQQQQPF	37	PQPQQPFPL	175
64	PQPQLPYPR	37	PFSQQQQPP	175
65	HQPQQQLPQ	36	LQPQLPYPQ	168
66	KQPQQPFPQ	36	SQPQQQSQQ	164
67	PQPQLPYSQ	36	FQPQQPYPQ	163
68	PFPQQPYPR	36	PQPQLLYPQ	152
69	PFPQPQQPY	35	PISQQQQQQ	151
70	PQPQQQFPQ	32	QFPQTQQPQ	144
71	QFPQQQFPQ	32	PFLQPQQPF	144
72	PQPQQIFPQ	32	PQQQLPQPQ	135
73	PFPQQQQHQ	31	MQPQQPFPQ	132
74	PYPQPQLPY	31	PQPQQPPQQ	130
75	PQPQQTFPQ	31	PQPFSQQPQ	130
76	PFPQPQLPY	30	IQPQQPAQY	124
77	QQQQQQQPQ	29	PQPQQPFSQ	120
78	PFPQQQQPL	28	QQPQQQFPQ	120
79	RQPQQPFPQ	28	QFSQPQQPQ	120
80	PFPQQQQPV	28	PFRQPQQPF	120
81	PQPQQQLLQ	27	QQQQQQQPF	117
82	QFPQQQQPL	26	QQPQQPLPQ	117
83	QQQQQPFPQ	26	HQPQQQVPQ	108
84	SQPQQQQPQ	26	RQPQQPFYQ	108
85	LQPQQQFPQ	26	QQPQQIFPQ	104
86	PQPQQPQQQ	25	IQSQQQQPQ	96
87	QQQQQQLPQ	24	FLPQLPYPQ	91
88	PFPQPQQAQ	24	QFPQQQIPV	86
89	VFPQPQQPQ	23	PQPQQTFPH	85
90	PFPQQQQQQ	23	LQPQQPYPQ	82
91	HQPQQPYPQ	23	HQPQQQFPQ	81
92	QRPQQPFPQ	22	PFPQPQLPF	80
93	PQQQQQLPQ	22	QQAQLPFPQ	78
94	QQPQQPFPL	22	QLPFPQQPQ	78

95	QQPQQPCPQ	21	QQPYPQQPY	77
96	PTPQQQFPQ	21	QFPGQQQPF	77
97	PFPQPQLPF	20	PQPQQPYSQ	75
98	PFSQPQLPY	20	PRPQLPYPQ	74
99	IQPQQQQPQ	20	QQSFPQQQQ	71
100	PQPQLLYPQ	19	QFPQQQQPL	69
